# Structural Characterization of Murine Phosphodiesterase 5 Isoforms and Involvement of Cysteine Residues in Supramolecular Assembly

**DOI:** 10.3390/ijms24021108

**Published:** 2023-01-06

**Authors:** Mauro Giorgi, Adriana Erica Miele, Silvia Cardarelli, Alessandra Giorgi, Mara Massimi, Stefano Biagioni, Michele Saliola

**Affiliations:** 1 Department of Biology and Biotechnology “C. Darwin”, Sapienza University of Rome, Piazzale A. Moro 5, 00185 Rome, Italy; 2 Department of Biochemical Sciences, Sapienza University of Rome, Piazzale A. Moro 5, 00185 Rome, Italy; 3 UMR 5280 ISA-CNRS-UCBL, Université de Lyon, 5 Rue de La Doua, 69100 Villeurbanne, France; 4 Department of Life, Health and Environmental Sciences, University of L’Aquila, Via Vetoio, 67100 L’Aquila, Italy

**Keywords:** PDE5, SAXS, TEM, MS, native-PAGE, quaternary structure, reactive Cys

## Abstract

Phosphodiesterases (PDEs) are a superfamily of evolutionarily conserved cyclic nucleotide (cAMP/cGMP)-hydrolyzing enzymes, components of transduction pathways regulating crucial aspects of cell life. Within this family, the cGMP-dependent PDE5 is the major hydrolyzing enzyme in many mammalian tissues, where it regulates a number of cellular and tissular processes. Using *Kluyveromyces lactis* as a model organism, the murine PDE5A1, A2 and A3 isoforms were successfully expressed and studied, evidencing, for the first time, a distinct role of each isoform in the control, modulation and maintenance of the cellular redox metabolism. Moreover, we demonstrated that the short N-terminal peptide is responsible for the tetrameric assembly of MmPDE5A1 and for the mitochondrial localization of MmPDE5A2. We also analyzed MmPDE5A1, A2 and A3 using small-angle X-ray scattering (SAXS), transmission electron microscopy (TEM), structural mass spectrometry (MS) and polyacrylamide gel electrophoresis in their native conditions (native-PAGE) and in the presence of redox agents. These analyses pointed towards the role of a few specific cysteines in the isoforms’ oligomeric assembly and the loss of enzymatic activity when modified.

## 1. Introduction

Second messengers, such as cyclic nucleotides (cyclic guanosyl- and cyclic adenosyl-monophosphate, cGMP and cAMP), are crucial factors regulating several processes of cells and tissues. In eukaryotes, the levels of cyclic nucleotides are regulated through the interplay of opposing enzymatic functions, namely nucleotide cyclases and phosphodiesterases (PDEs). Mammalian PDEs are classified in eleven structurally related gene families, where family number five is specifically dedicated to cGMP hydrolysis (cyclic 3′,5′-GMP phosphodiesterase, EC 3.1.4.35). Moreover, within each family, multiple gene promoters, alternative splicing and post-translational modifications contribute to increasing the variability and molecular diversity as well as the tissue and cellular distribution [1,2,3,4,5]. Mammalian PDE5 is a known regulator of vascular smooth muscle contraction, and its deregulation has been linked to cardiovascular diseases, cancer, memory loss and cytokine-storm-associated inflammation [6,7,8,9,10,11,12]. In fact, PDE5 is the molecular target of several well-known drugs used to treat erectile dysfunction, pulmonary hypertension and cardiovascular diseases [6]. In recent years, we have focused our attention on the three isoforms of *Mus musculus* PDE5. The murine recombinant isoforms of PDE5A1, A2 and A3 were individually cloned, were expressed in the model yeast *Kluyveromyces lactis* and were purified through affinity and size exclusion chromatography [13,14]. The three isoforms were almost identical, differing only at their N-terminus. A1 and A3 were translated from the same mRNA from two different start codons; therefore, A1 had an N-terminus that was 41 amino acids longer than that of A3. Isoform A2 originated from an alternatively spliced mRNA, and it had 10 more amino acids than A3 (Table 1). It must be emphasized that the N-terminus of PDE5 isoforms is highly conserved among mammalian species, suggesting an evolutionarily conserved role of these N-terminal peptides [15].

Recently, we demonstrated that this extra sequence was able to direct the translocation of MmPDE5A2 into the mitochondria in both yeast and mammalian cells, whereas the localization of the other two isoforms remained cytosolic and/or nuclear [15,16]. In addition, we showed that the overexpression of MmPDE5A1 and A3 induced a fermentative metabolism in the *K. lactis* yeast, while MmPDE5A2, which has a mitochondrial localization, is mutagenic and was able to induce a Rag-phenotype (Resistance to antibiotics on glucose) in the host, i.e., a rag mutation that rendered the host cells unable to grow in the presence of mitochondrial inhibitors [17,18].

Moreover, we provided evidence that the distinct localization of MmPED5A2 compared to MmPDE5A1 and MmPDE5A3 in the milk yeast affected the intracellular redox balance as a consequence of the metabolic imbalance, oxidative vs. fermentative [16].

In this paper, we attempted to give a structural explanation of this metabolic effect by means of small-angle X-ray scattering (SAXS), transmission electron microscopy (TEM), structural mass spectrometry (MS) and polyacrylamide gel electrophoresis in native conditions (native-PAGE) and in the presence of redox agents.

## 2. Results

### 2.1. Quaternary Structure of MmPDE5A1, A2 and A3

The murine recombinant isoforms of PDE5A1, A2 and A3 were expressed in *K. lactis* and purified through affinity and size exclusion chromatography [13]. As previously reported, purified MmPDE5A1, when fractioned in native PAGE, displayed different conformations, called D1, D2 and D3, in the presence of substrates, inhibitors and thiol compounds [14,16,19,20,21]. Using this technique in combination with the size exclusion chromatography (SEC) and SAXS analyses, we showed that this longer isoform (Table 1) could also assemble into flexible tetramers, which we called T1, T2 and T3 [14]. Moreover, we had previously shown the ability of platelet-derived PDE5A1 to behave in the same way, demonstrating that the tetramer was not an artifact of MmPDE5A1 overexpression in a heterologous host [14]. Moreover, the platelet-derived protein also formed higher-order assemblies.

We decided to use this same set of experiments, i.e., native PAGE and SEC-SAXS analyses, on all three isoforms in order to infer the role of the N-termini in the quaternary structure as well as their effect on cellular localization and on the metabolic imbalance.

**Figure 1:**
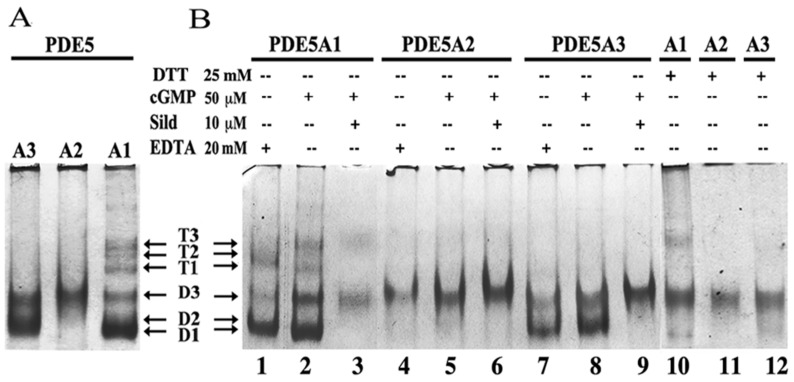
Native PAGE of MmPDE5 isoforms. (**A**) Migration mobility of freshly purified MmPDE5 isoforms and (**B**) that after incubation for 1 h at 30 °C with substrate (cGMP), inhibitor (sildenafil, Sild), bivalent cation chelator (EDTA) and a reducing agent (dithiothreitol, DTT) at the concentrations specified in the figure. Bands were visualized through Coomassie staining.

Indeed, the three isoforms behaved differently in native PAGE (Figure 1) and in the SEC-SAXS analyses.

As shown in Figure 1A, MmPDE5A1 assembled into very flexible dimers (D1-D2-D3) and tetramers (T1-T2-T3) as previously reported [13,14]. MmPDE5A3 only assembled into dimers; though, they were quite flexible (D2-D3). Meanwhile, MmPDE5A2 displayed a unique and compact quaternary structure (D3). Moreover, as can be seen in Figure 1B, while the A1 and A3 isoforms underwent some conformational rearrangements with diverse ligands (lanes 1, 2, 3, 10 and 7, 8, 9 and 12, respectively), A2 was unable to modify its mobility with either cGMP, the inhibitor (sildenafil), the chelator (EDTA) (lane 4–6) nor the reducing agent (DTT) (lanes 4, 5, 6 and 11).

**Figure 2:**
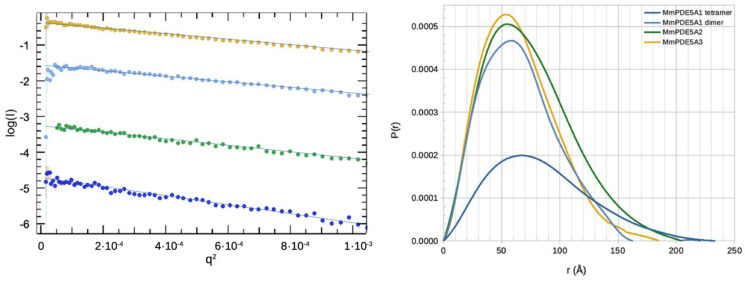
Summary of SAXS analysis. Left panel: Guinier analysis of I(q) as a function of q^2^ of deconvoluted data. From top to bottom, there are MmPDE5A3 (orange), MmPDE5A2 (green), MmPDE5A1 dimer (light blue) and MmPDE5A1 tetramer (dark blue). Right panel: GNOM analysis of the probability function after deconvolution.

To further characterize the quaternary structure of the three MmPDE5 isoforms, we analyzed them at the SOLEIL synchrotron facility (St Aubin, France) through small-angle X-ray scattering coupled with size exclusion chromatography (SE-HPLC-SAXS) with the SWING beamline [22]. The protein solutions were loaded on S200 5/150 prepacked columns (Cytiva Healthcare) and were eluted under isocratic flux at 0.2 mL/min at 20 °C. Less than 5% of each isoform was aggregated and eluted with the dead volume. Indeed, the sequence differences in the N-terminal region determined a different quaternary organization of the MmPDE5 isoforms in solution. Namely, A1 was confirmed to be 40% in tetrameric form and 60% in dimeric form as previously reported [14] and as the gaussian deconvolution in US-SOMO [23] sorted out; however, A2 and A3 were dimers. The analysis with both Scåtter [24] and US-SOMO [23] gave concordant overall radius of gyration (Rg) and maximum dimension of the particle (Dmax) values (Table 2, Figure 2) as well as those of the gaussian deconvoluted values of the tetramer and dimer (Table 2). Moreover, the data extracted from the main elution peak of each isoform were reasonably fitted using the coordinates of the closest homologue found in the PDB (human PDE2 3IBJ, [25]), giving χ^2^ = 0.99 for A3, χ^2^ = 1.14 for A2 and χ^2^ = 1.51 for A1. The relative amounts of dimers and tetramers found using the program Oligomer from the ATSAS suite [26], using a model of the tetramer based on our previously published data [14], were consistent with those found using the gaussian deconvolution of the scattered peaks with US-SOMO [23] and using SVD with ScÅtter [24].

The flexibility analysis of the main elution peaks of each isoform before deconvolution highlighted another characteristic, namely the flexibility of the isomers. In fact, MmPDE5A1 was the most heterogeneous isoform, ranging from tetramers to dimers, where each assembly was very flexible, confirming both our previous analysis [14] and what was seen in the native PAGE (Figure 1A). MmPDE5A2 and A3, on the contrary, seemed to be more rigid although some degree of flexibility was still present, possibly explaining the differences in the Dmax values (Figure 2, right panel).

**Figure 3:**
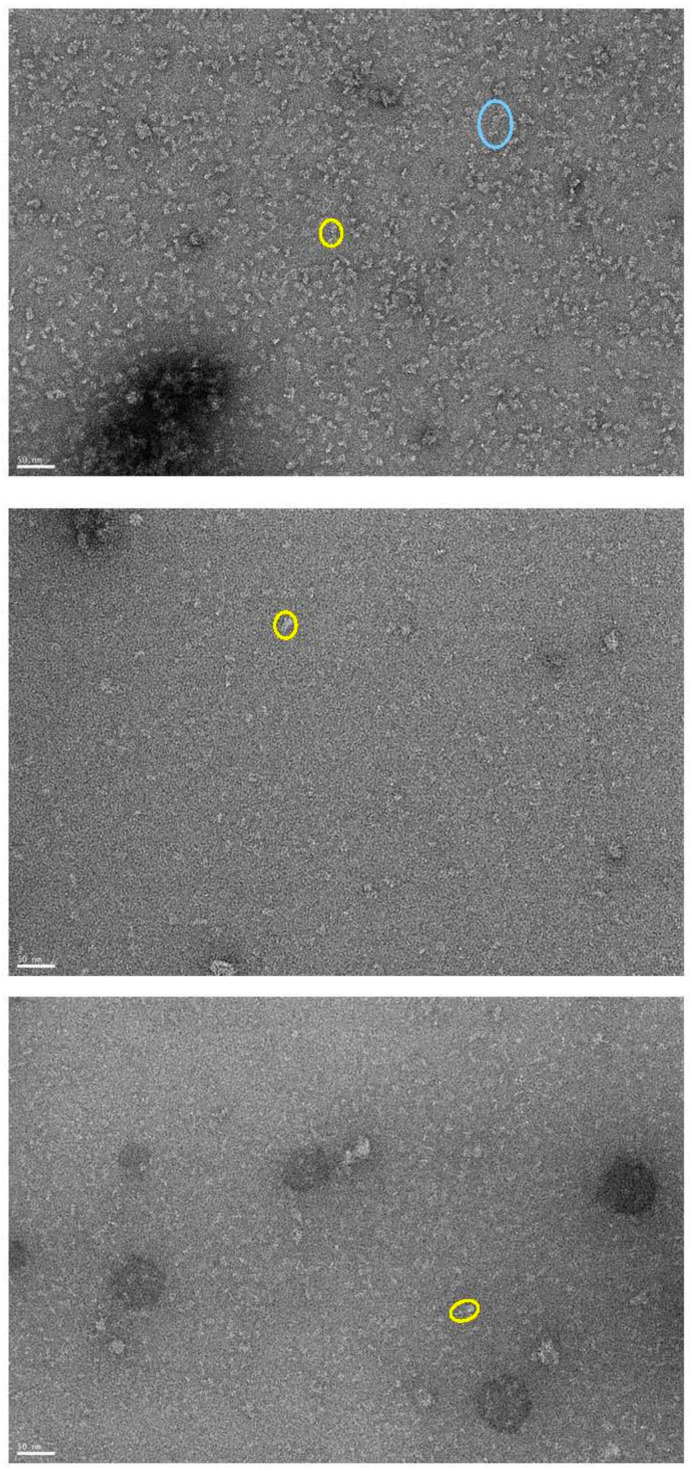
TEM pictures of MmPDE5A1 (**top**), A2 (**center**) and A3 (**bottom**) after negative staining and in the absence of ligands/effectors. In the cyan circle, there is one representative particle of tetramers; in the yellow circles, there are representative dimers. The scale bar is 50 nm, and magnification was 30,000×. See Section 4.3 in methods for details.

The purified isoforms of MmPDE5 were also analyzed through negative-stain transmission electron microscopy (TEM). As expected, MmPDE5A1 was a mixture of tetramers (cyan circle) and dimers (yellow circle), but there were also larger aggregates. Aggregates were also present in the A2 and A3 isoforms together with the dimers (Figure 3). The MmPD5A2 and A3 particles in the images (yellow circle) displayed an elongated shape of approximately 190 Å long and 60 Å wide, not far from the dimensions of the close cAMP-dependent PDE2, which we used to fit the dimer in the SAXS data (3ibj, [25]). Moreover, the tetrameric assembly was compatible with a head-to-head configuration, suggesting a role of the N-terminal extra peptide in reaching this conformation.

### 2.2. Structural Effects of Reducing and Oxidizing Agents

**Figure 4:**
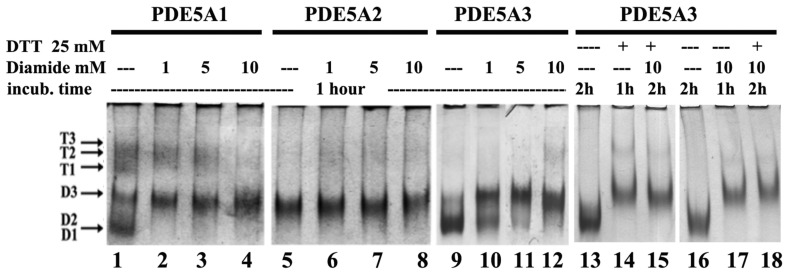
Migration mobility of PDE5 isoforms in native PAGE after incubation for 1 h at 30 °C in the presence of increasing concentrations of either diamide or diamide and DTT (or the opposite order) added in succession after 1 h and incubated for one more hour. Each lane contained 1 μg of purified MmPDE5A1, A2 and A3. Proteins were visualized through Coomassie staining.

Since we had highlighted a role of the three isoforms in the regulation of the redox metabolisms in both *Saccharomyces cerevisiae* and *K. lactis* [16,21] and in light of the effect of the reducing agent in native PAGE (Figure 1B, lanes 10, 11 and 12), we decided to investigate in depth the effect of external redox agents on their quaternary structure and on their hydrolyzing activity. Our attention was centered on the possible role of cysteine residues since it had already been shown that the in vivo activity of PDE could be modulated with Ellman’s reagent (5,5-dithio-bis-(2-nitrobenzoic acid), DTNB) and dithiothreitol (DTT). In the primary structure, PDE5 contains 25 Cys per monomer. Therefore, the three isoforms were incubated in the presence of a large excess of reducing or oxidizing agents to determine the effect on their quaternary structure. To this end, the purified MmPDE5A1, A2 and A3 were first incubated in the presence of diamide, a reagent that oxidizes sulphydryl groups to disulfides [27], and their migrating properties were analyzed in native PAGE. The results of this analysis (Figure 4) showed the disappearance of the tetrameric bands of MmPDE5A1 with increasing concentrations of diamide (lanes 1–4), and they showed the concomitant accumulation of the D3 migrating band for both PDE5A1 (lanes 1–4) and A3 (lanes 9–12). In contrast, the migrating properties of PDE5A2 were unmodified (Figure 4, lanes 5–8).

Addition of DTT alone (Figure 1B, lanes 10–12) rigidified A1 and A3 towards T3 and D3 in the former and D3 in the latter. The addition of diamide was more disruptive for A1, and it was concentration dependent. MmPDE5A2 remained stable at D3 independently of the reducing or oxidizing agents, even when added in large excess.

These results indicated that neither diamide, an oxidizing agent, nor DTT, a reducing agent, were able to modify the rigid dimer of MmPDE5A2. At the same time, both reagents were able to induce a D3 conformation in A1 and A3, indicating that Cys modification impacted the quaternary assembly, whatever the pathway. Keeping in mind that A2 is translocated into the mitochondria and purified from there, we might hypothesize that the presence of intramitochondrial modifiers would be able to irreversibly rigidify this isoform.

In order to better understand the role of diamide vs. DTT, we incubated MmPDE5A3 in succession one after the other to test whether they differently affected its native PAGE migration properties. Surprisingly, the order did not matter with respect to inducing the D3 conformation (Figure 4, lanes 13–18). It is worth reminding that the conformation of D3 was also the main outcome of the binding of the substrate and inhibitor (cGMP and sildenafil, Figure 1B). Putting all these data together, we could conclude that Cys modification and ligand binding converged towards a thermodynamically preferred rigid D3 conformation.

### 2.3. Characterization of Cys Involved in D3 Stabilization

**Figure 5:**
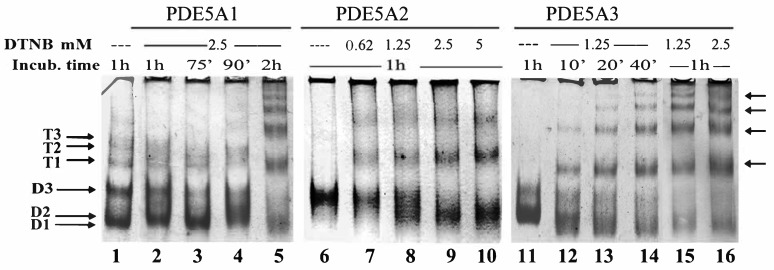
Native PAGE migration mobility of PDE5 isoforms incubated at different times in the presence of increasing concentrations of DTNB. Arrows indicate higher-order oligomers larger than tetramers. Each lane contained 1 μg of purified MmPDE5A1, A2 and A3. Proteins were visualized through Coomassie staining.

In order to explore in depth the Cys residues responsible for inducing the D3 conformation, we decided to use DTNB to spectrophotometrically titrate the exposed Cys, which could possibly be physiologically relevant [28]. Before titration, we explored several concentrations of the reagent and several incubation times, and we used native PAGE to visualize the effects on the quaternary structures and conformations (Figure 5). Much to our surprise, DTNB affected the three isoforms differently than DTT and diamide; nevertheless, the absence of smears indicated a specific effect. At the end of the long incubation times, all the isoforms formed higher-order oligomers, including MmPDE5A2. Moreover, a convergence of the conformations towards D1 and T1 instead of D3 was apparent and was reproduced many times (Figure 5 represents one of the many native PAGE runs of several purification batches).

Furthermore, we noticed that MmPDE5A1 was somehow more resistant to the formation of higher-order oligomers compared to A2 and A3. This latter isoform started to multimerize after 10 min incubation with 1.25 mM DTNB, while A2 became tetrameric after 1 h incubation with 0.62 mM DTNB. Altogether these data showed that: (i) the tetramer, when present, protected the protein from the multimerization induced by DTNB; (ii) the dimers were more sensitive to crosslinking; and (iii) DTNB was able to remove the rigidity of A2 through the modification of its Cys.

**Figure 6:**
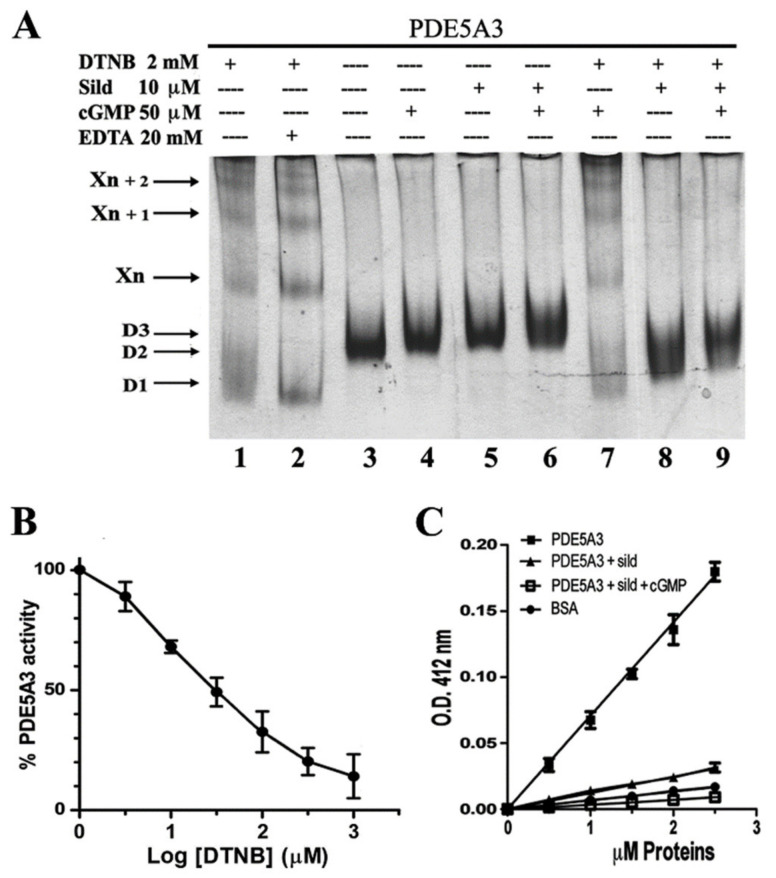
Characterization of Cys reactivity in MmPDE5A3. (**A**) Native PAGE migration of the isoform following incubation at 30 °C for 1 h with DTNB, cGMP and sildenafil. EDTA, cGMP and sildenafil were preincubated for 1 h prior to addition of DTNB and were followed by 1 extra hour of incubation. (**B**) Inhibition of MmPDE5A3 activity with increased amounts of DTNB. (**C**) Titration of cysteine thiols of PDE5A3 isoform alone with sildenafil or sildenafil plus cGMP using DTNB. Bovine serum albumin (BSA) thiol groups were also titrated as a control.

In order to better characterize the role of the Cys, we decided to focus on MmPDE5A3 alone since it represents the minimal common part of the three isoforms (Table 1), it is a flexible dimer and it is sensitive to external ligands. We, therefore, performed the incubation with DTNB in the presence of cGMP and Sildenafil, respectively the substrate and one known inhibitor. As can be seen in Figure 6A, by comparing Figure 1B lanes 7–9 and Figure 5 lanes 11–12, the quaternary structure of MmPDE5A3 was affected by DTNB, formed tetramers and higher-order oligomers (marked as Xn + 1, etc.) independently of the presence of ligands and divalent cations (Zn^2+^ and Mg^2+^), which are needed for activity (Figure 6A, lanes 1, 2 and 7). Thus, EDTA and cGMP did not counteract the multimerization induced by DTNB. On the other hand, the presence of the inhibitor alone or in combination with the substrate was able to block the action of DTNB (Figure 6A, lanes 8 and 9). Indeed, sildenafil together with cGMP seemed to induce the D3 conformation better than single incubation with just the inhibitor, and, somehow, they locked it and prevented further Cys modifications. The inability of the cGMP alone to contrast DTNB might be due to its hydrolysis during the 1 h incubation prior to DTNB addition.

Given the presence of oligomers and since DTNB is known from the literature to be an inhibitor of PDE [29], we measured the hydrolyzing activity of MmPDE5A3 in the presence of increasing concentrations of the Ellman’s reagent from 1 μM to 1 mM (Figure 6B). Indeed, the activity was drastically reduced up to a residual of about 17%, which we ascribed to the residual dimer (lowest band in lane 1 in Figure 6A). The calculated IC_50_ was about 30 μM.

Finally, we quantified the number of Cys able to react with DTNB (Figure 6C). According to the assay, the number of -SHs specifically involved in the binding of DTNB was 5.05 ± 0.06 per each MmPDE5A3 monomer. We repeated the assay in the presence of the inhibitor sildenafil, and the number of thiols that were oxidized decreased to 0.87 ± 0.02 per each subunit. This result indirectly showed that the reactive Cys were close to the active site and were shielded by the inhibitor; therefore, they became unable to react with DTNB. Furthermore, the assay carried out in the presence of both the inhibitor and substrate gave a very low number of reactive thiols (0.22 ± 0.03 per each subunit), suggesting that the concomitant occupancy of both the active site by the former and the allosteric site by the latter almost completely blocked the oxidation of Cys by DTNB. This was in line with the qualitative results obtained by native PAGE (Figure 6A).

As a control, we titrated the number of reactive thiols in bovine serum albumin (BSA) and found 0.477 ± 0.006 units per monomer, a value that was in good agreement with the literature data [30]. From the sequence, we knew that there were nine Cys in the catalytic domain, nine in the regulatory domain GAF-A and seven in the regulatory domain GAF-B. Out of these twenty-five, only five were titrated in the absence of the inhibitor, one was titrated in its presence and almost none were titrated when both sildenafil and cGMP were present; hence, we may say that at least four reactive Cys were in the catalytic domain and that at least one was in the allosteric site.

### 2.4. Effect of the Covalent Modification of Cys on the Quaternary Assembly of MmPDE5A3

In order to rationalize the results obtained with DTNB and to compare them to those obtained with diamide and DTT, we used the alkylating agent N-Ethyl-Maleimide (NEM), which covalently blocks unmodified thiols. This reagent is widely used to detect solvent-exposed Cys residues possibly involved in quaternary assembly and/or in physiopathological processes [31].

**Figure 7:**
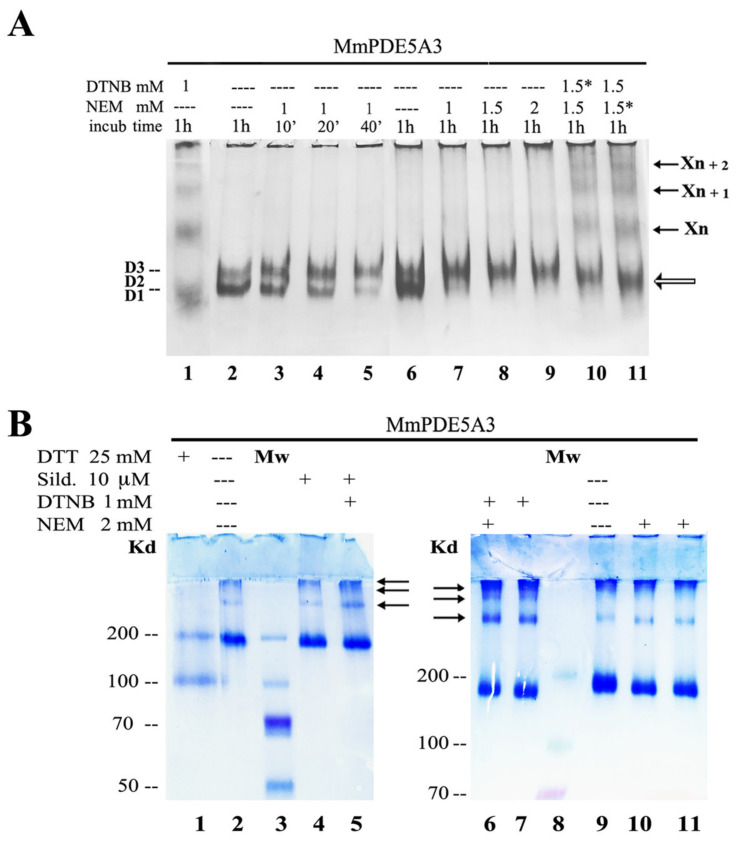
Effect of NEM on MmPDE5A3 visualized through native- and SDS-PAGE. (**A**) Migration of MmPDE5A3 on native gel in the presence of DTNB and NEM alone or in combination. The chemical marked with * was added 30 min after the other chemical. (**B**) Different migration of unboiled samples of MmPDE5A3 alone or after incubation with DTT, sildenafil, DTNB and NEM on denaturing, not reducing, SDS-PAGE. MW = molecular weight markers. The black arrows indicate the oligomers formed after incubation with DTNB (compatible with tetramers, hexamers and octamers).

Figure 7A shows the migration mobility of MmPDE5A3 in native PAGE subjected to increasing concentrations of NEM (lanes 8–10) or progressively longer times of incubation with NEM alone (lanes 3–6) and in combination with DTNB (lanes 11–12).

NEM did not induce oligomerization as DTNB did; on the contrary, the behavior was similar to that induced by DTT and diamide (Figure 7A vs. Figure 1 and Figure 4). At the same time, it did not prevent DTNB from promoting oligomerization independently of the order of incubation. These results suggested that different Cys residues were available to be modified by either NEM or DTNB.

As a control and to further evidence the presence of high-molecular-mass oligomers, we also ran the same samples on an SDS-PAGE without boiling them nor adding reducing agents in the loading buffer (Figure 7B). Under these conditions, the protein was, for the large majority, a dimer (lanes 2 and 9), while the monomer and the dimer were present in lane 1, as an excess of DTT was incubated with the A3 isoform. Nevertheless, a small amount of high-molecular-weight species was present under these conditions in both the control and in the presence of the inhibitor (Figure 7B, lanes 2, 4 and 9). These migrating species were slightly increased in the presence of both the inhibitor and DTNB (Figure 7B, lanes 5), while, at the same time, the amount and number of these oligomers were greatly increased with DTNB with/without NEM (see the arrows of lanes 6–7).

### 2.5. Localization of Reactive Cys Using Mass Spectrometry (MS)

In order to identify the Cys residues capable of inducing oligomerization, we performed MS analysis of the tryptic peptides of MmPDE5A3 before and after the modification of the Cys with NEM. This analysis was able to map twenty-three out of twenty-five Cys residues (Supplementary Figures S1–S3) and highlighted six highly reactive Cys, of which three were already oxidized to Cys-sulfenic acid before any treatment, indicating their high intrinsic reactivity. In particular, Cys 258, 315 and 711 were 100% modified by NEM. The peaks of the peptides corresponding to Cys 117, 447, 522 and 544 appeared in the presence of NEM; though, they also presented a minor signal with iodoacetamide (IAA). Moreover, Cys 447, 522 and 544 were already oxidized to Cys-sulfenic acid in the untreated sample. Based on these data, we tried to localize those Cys on a 3D structure modeled using the isolated domains present in the protein data bank (PDB). At present, the structure of the isolated GAF-A of MmPDE5A1, from residue 154 to 302 (PDB ID: 2k31), the structure of the GAF-B of human PDE5 (residues 307–488, PDB: 3xss), more than 70 structures of the catalytic domain of human PDE5 (aa 537–858, we used PDB: 5jo3) and the structure of the homologous cAMP hydrolyzing PDE2 (almost the full length, PDB: 3ibj) are available. According to the model, Cys 117 and 258 belong to the regulatory domain GAF-A and are exposed to the solvent, Cys 315 and 437 belong to the second regulatory domain GAF-B and are shielded from the solvent, while Cys 522, 544 and 711 belong to the catalytic domain and are buried close to the Zn^2+^ coordination site (Supplementary Table S1).

## 3. Discussion

PDEs, strictly defined as 3′-5′ cyclic nucleotide phosphodiesterases, are a superfamily of enzymes that modulate the amplitude and duration of cyclic nucleotide signaling through their hydrolysis to AMP and GMP [1,2]. Using *K. lactis* as a model organism, the full-length isoforms of cGMP-dependent murine phosphodiesterase 5 (MmPDE5A1, A2 and A3) were successfully cloned, purified and studied, evidencing, for the first time, a specific role of each isoform in the control, modulation and maintenance of the cellular metabolism [13,14,16,21].

The three isoforms were almost identical, only differing at their N-terminus. In fact, MmPDE5A1 displayed an N-terminal peptide that was 41 amino acids longer than A3 (Table 1). This peptide proved to be an extra element of regulation, allowing the assembly of PDE5A1 also into tetramers, besides the dimer, in both yeast and rat platelets [14]. PDE5A2 had ten more amino acids than A3, a peptide capable of directing the translocation of this activity into the mitochondria, whereas the localization of the other two isoforms was most likely cytosolic or nuclear [16]. PDE5A3, being devoid of the extra N-terminus, shared with the other two isoforms the common identical part of the protein (i.e., 823 amino acids, Table 1).

Many reports, including that from our group, have previously shown that PDE5 dimers and tetramers can assume in native PAGE several conformational forms determined by the binding of ligands to both the catalytic and allosteric sites. [14,16,19,20].

In this paper, the three MmPDE5 isoforms were further characterized, showing that MmPDE5A1 assembled into a mixture of dimers and tetramers which were both quite flexible as demonstrated through SAXS and negative-stain single-particle TEM. MmPDE5A2 and MmPDE5A3 were dimers; though, the former was quite rigid and insensitive to most ligands, and the latter was as flexible as the dimers of MmPDE5A1. All of them tended to form high-molecular-weight aggregates when the concentration exceeded 20 μM, which was in line with what was shown in platelet-derived PDE5 [14].

These structural and conformational modifications were also induced through treatment with the reducing and oxidizing agents acting on Cys residues.

Diamide, DTT and NEM had the same impact on the MmPDE5 isoforms: they rigidified the quaternary structure of A1 and A3 (Figure 1, Figure 4 and Figure 7), but they had no effect on A2, possibly because the mitochondrial localization of this isoform put it into contact with endogenous Cys modifiers. Overall, these results were somehow unexpected given the nature of the three reagents. In fact, diamide is a reagent that oxidizes free sulphydryl groups to disulfide [27]; DTT reduces disulfide and mixed dithiol (S-S, S-NO, S-SG and SOH) to free SH thiols [32]; and NEM is an alkylating agent acting on reactive free thiols [31].

Therefore, in an attempt to deepen the understanding of the role of Cys, we decided to titrate the free exposed ones with Ellman’s reagent, DTNB [33]. We performed the titration on MmPDE5A3 given that it contained the common 823 residues, including 25 Cys, and that there were no Cys in the N-terminal peptides of A1 and A2.

Indeed, DTNB disrupted the canonical conformations of the three purified PDE5 isoforms, as evidenced in native PAGE (Figure 5), leading to the progressive accumulation of higher-order oligomers (Figure 5) and to the progressive loss of catalytic activity (Figure 6B). Moreover, the preincubation of the sample with sildenafil alone or together with cGMP prevented modification and blocked oligomerization (Figure 6A).

These results indicated a protective effect of the substrate and inhibitor on the cysteine residues specifically targeted by DTNB. In fact, the number of reactive thiols titrated on PDE5A3 was about 5/monomer. The addition of sildenafil prior to DTNB reduced the reactive thiols to about 1/monomer, while the combined action of the substrate and inhibitor protected practically all the -SH groups from DTNB (Figure 6C), which was in agreement with the data of Figure 6A.

However, NEM was unable to block the higher-order oligomeric structures induced by DTNB if they were incubated one after the other, suggesting that the thiols targeted by DTNB were not the same as those covalently blocked by NEM (Figure 7A).

When we carried out the identification through MS of NEM-modified Cys residues, we found seven putative candidates, namely Cys 117 and 258, belonging to the regulatory domain GAF-A and being exposed to the solvent; Cys 315 and 447, belonging to the regulatory domain GAF-B and being buried; and Cys 522, 544 and 711, belonging to the catalytic domain and being close to the Zn^2+^ binding site and active site. Hence, we hypothesized that the Cys in the GAF-A domain were the most probable candidates to activate the formation of concatemers (indicated by X_n_, X_n+1_ and X_n+2_ in Figure 7A), while the Cys in the catalytic domain were those that were totally prevented from reacting when the inhibitor was present.

Indeed, as suggested by [34], GAF-A underwent a major conformational change upon cGMP binding or, as in our case, DTNB attack, thus affecting the overall conformation of the PDE5 holoenzyme. Therefore, this event might lead to the progressive oxidation of other residues in the catalytic site with the accumulation of higher-order oligomers and the loss of activity. It is worth mentioning that a predicted model by AlphaFold of the MmPDE5A1 monomer is present on the UniProt webpage (https://www.uniprot.org/uniprotkb/Q8CG03/entry (accessed on 23 March 2022)). In this model, the first one hundred amino acids are predicted to be intrinsically disordered and are bent towards the catalytic domain, exposing the Cys 258 in the GAF-A domain even more, which opens up with respect to the compact dimer of its homologue in the structure of PDE2 (Supplementary Table S1). Moreover, in the TEM images, the particles were highly dynamic, preventing a clear 2D classification and, hence, a 3D structure reconstruction.

By and large, these results suggested that the modular architecture of PDE5 was built to better respond to the attack of many different reagents, rigidifying its quaternary structure in a few conserved conformations and building higher-order oligomers which could switch off its catalytic activity. We hypothesized that the Cys in the GAF-A domain were the most probable candidates to activate the addition of dimers to the concatenamer (X_n_, X_n+1_, etc.), while the Cys in the catalytic domain were those which were totally prevented from reacting when a ligand or an inhibitor was present. Moreover, since DTNB inhibition did not lead to a complete loss of activity (Figure 6B) nor to a complete disappearance of the dimer (Figure 6A), we suggested that the higher-order oligomerization, determined by DTNB, may be reversible under physiological conditions. It is worth reminding that PDE5 links cGMP signaling to NO signaling and that both are linked to cellular redox stress sensing [4,8,35]; therefore, it is not unreasonable that the modulation of its function and structure happened through specific Cys modifications. Finally, we wondered which was the physiological event that led to the supramolecular assembly/loss of PDE activity. Previously [14,21], we showed that the cAMP/cGMP balance was the determinant factor on the basis of the control and modulation of the cellular metabolism/maintenance of the redox balance. We suggested that the progressive accumulation of inactive multimers associated with the reduced hydrolysis of cGMP might be an in vivo cellular physiological mechanism that occurs to respond to this imbalance and to restore cyclic nucleotide homeostasis. Of course, this unbalance may be related to several pathologies, such as those reported in the literature [1,2,4,6,7,9,10,11,12].

## 4. Materials and Methods

### 4.1. Strains, Media and Vectors

The genotype of *Kluyveromyces lactis* CBS2359 strain (www.cbs.knaw.nl (accessed on 12 March 2018)), the media, the growth conditions and all the other materials and protocols used for the heterologous production, affinity purification and enzymatic characterization of the recombinant full-length MmPDE5A1, A2 and A3 isoforms were previously described [13]. Detailed cloning of PDE5A1, A2 and A3 spliced variant genes fused to 3XFLAG tag in *K. lactis* pYG137/1 vector was previously described [13].

### 4.2. Size Exclusion Chromatography Linked to Small-Angle X-ray Scattering (SEC-SAXS)

SAXS measurements were performed at the SWING beamline of the SOLEIL synchrotron facility (St Aubin, France) [22]. Data collection was performed at 293 K, 12.5 keV, 1 frame/s and 15% beam transmission (I = 500 mA; I_0_ = 1.26 × 10^−7^; I_t_ = 4.147 × 10^−6^). Data were recorded on an Eiger 4M (Dectris AG, Baden-Daettwil, CH) placed at 2000 mm of distance. Size exclusion chromatography was performed on an HPLC system (Shimadzu France, Marne La Vallée, F) in line with the SWING beamline, and 2 samples per each isoform were automatically injected (40 μL at 3 and 1 mg/mL) into a Superdex S200 5/150 (Cytiva Europe GmbH, Velizy-Villacoublay, F) previously equilibrated with 50 mM HEPES pH 7.5, 75 mM NaCl and 37.5 mM MgCl_2_; the column was run at 293 K with a constant flow of 0.2 mL/min, and 1 image was collected. The 2D scattering images were converted into 1D profiles with FoxTrot (Xenocs SAS, Grenoble, F); the scattering profiles were analyzed with US-SOMO-SAXS module [23] and Scåtter [24]. The gaussian deconvolution algorithm in the first program and the SVD deconvolution in the second program were used to extrapolate the relative proportion of dimers and tetramers in solution. The radius of gyration (Rg) and the maximal dimension (Dmax) of the scattering particles were derived through Guinier analysis in both programs (Table 2, Figure 2).

### 4.3. Transmission Electron Microscopy

Images of MmPDE5A1, A2 and A3 were collected at the IBS electron microscope facility of the Grenoble Instruct-ERIC center (France). The images were taken under low-dose conditions (<10 e-/Å^2^) with defocus values between 1.2 and 2.5 μm on a Tecnai 12 LaB6 electron microscope at 120 kV accelerating voltage using CCD Camera Gatan Orius 1000. A total of 5 μl of each sample was diluted to 0.6 mg/mL, was absorbed into the clean side of a carbon film on mica, was stained with uranyl acetate (UO_2_(CH_3_COO)_3_·2H_2_O at 2% in distilled water (pH 4.2–4.5)) for 5 s and was transferred to a 400-mesh copper grid.

### 4.4. PDE Enzymatic Assays

PDE activity was measured at 30 °C with the two-step method described in [36] using [^3^H]cGMP (PerkinElmer, MA, USA). Aliquots of eluted fractions from SEC were incubated in 60 mM HEPES pH 7.2 assay buffer containing 0.1 mM EGTA, 5 mM MgCl_2_, 0.5 mg/mL bovine serum albumin and 30 μg/mL soybean trypsin inhibitor in a final volume of 0.15 mL. The reaction was started by adding tritiated substrate at a final concentration of 1 μM and stopped by adding 0.1 M HCl. The specific activity was quantified at the 10% limit of the total substrate hydrolyzed. Sildenafil was a generous gift from Pfizer. Protein concentration was determined according to [37].

### 4.5. Native Polyacrylamide Gel Electrophoresis (PAGE)

PAGE was performed with a 5% nondenaturing acrylamide gel in a Tris–glycine pH 8.3 running buffer at 4 °C for 60–80 min with a current of 20 mA in a Bio-Rad Mini-Protean electrophoresis apparatus [38]. Each well was loaded with 1.0 μg of purified recombinant MmPDE5A1, MmPDE5A2 or MmPDE5A3 and was preincubated at 30 °C with substrate and/or inhibitor/modifier at the concentrations and times specified in the figures; the activity buffer consisted of 5 μL of 50 mM HEPES pH 7.5, 50 mM NaCl and 15 mM MgCl_2_. Protein bands were visualized through Coomassie staining. The concentrations of sildenafil and cGMP were higher than therapeutically used concentrations in order to demonstrate the conformational structural changes according to [19].

### 4.6. Titration of Free SH Using Ellman’s Method

Different amount of MmPDE5A3 and BSA were adjusted to a final volume of 1 mL of assay buffer (20 mM HEPES pH 7.5, 25 mM MgCl_2_ and 75 mM NaCl) in the presence of 0.5 mM DTNB. After 1 h of incubation at room temperature, the sample was transferred in a disposable plastic cuvette (1 mL volume, 1 cm light path) to read the absorbance at 412 nm. To calculate the moles of titrated sulphydryl groups, we used a molar absorptivity of ε_412_ = 14,150 M^−1^ [39].

### 4.7. Mass Spectrometry Analysis

Electrophoretic bands corresponding to the protein “control” and the protein treated with N-Ethylmaleimide (NEM) were submitted to tryptic proteolysis. The bands, after some steps for destaining and dehydrating, respectively, with an aqueous solution of 50 mM ammonium bicarbonate with or without 50% of acetonitrile and pure acetonitrile were reduced with 10 mM dithiothreitol (DTT) first and then alkylated with 55 mM iodoacetamide (IAA). The proteolysis was carried out with 100 ng of trypsin in 25 mM ammonium bicarbonate at 37 °C overnight. The tryptic mixtures were analyzed with an UltrafleXtreme MALDI ToF/ToF (Bruker, Bremen DE, Germany) equipped with a smartbeam-II laser.

## 5. Conclusions

In conclusion, the extra N-terminal sequence of MmPDE5A1 is necessary and sufficient for assembly into dimers and head-to-head tetramers. That of MmPDE5A2 is sufficient for translocating it into the mitochondria [16], where it is possibly modified into a rigid dimer that is insensitive to ligands other than DTNB. The common part among the three isoforms that coincides with the entire MmPDE5A3 is able to form flexible dimers that respond to redox agents and form regulated multimers of two, possibly by interdimer disulfide bridge formation through the Cys in the regulatory GAF-A domain. However, some extra S-S bonds can also occur, which lead to the inactivation of the enzymatic activity. Given the conservation of the N-terminal extension in other mammalian PDE5 [15], we might predict a similar regulatory role in all the family members.

We believe that the present work will help to build a complete picture of the complex behavior and regulation of PDE5 by means of differently spliced isoforms, post-translational modifications, other than phosphorylation, and oligomerization induced by the redox state of the environment.

## Figures and Tables

**Table 1:** N-terminal amino acid sequences (1 letter code) of MmPDE5 isoforms. The bolded section is the start of the common sequence, and the UniProt ID is in parentheses (https://www.uniprot.org (accessed on 23 March 2022)).

MmPDE5A1 (Q8CG03)	MERAGPNSVR SQQQRDPDWV EAWLDDHRDF TFSYFIRATRD **MVNAWFSE-**
MmPDE5A2 (A0A0G2JF67)	MLPFGDKTRD **MVNAWFSE-**
MmPDE5A3	**MVNAWFSE-**

**Table 2:** Summary of SAXS data analysis. The fraction of aggregated proteins, dimeric and tetrameric forms, were derived through gaussian deconvolution of the scattered curves dI(q)/dt with the US-SOMO HPLC-SAXS module [23]. Then, the corresponding I(q) vs. q curves of each deconvoluted peak were analyzed individually to retrieve the Rg and Dmax values.

Isoform	Rg	Dmax	% Aggregation	% Tetramer	% Dimer
MmPDE5A1	66 Å (T) 55 Å (D)	235 Å (T) 160 Å (D)	1.5	40	60
MmPDE5A2	55 Å	202 Å	5	3	97
MmPDE5A3	53 Å	185 Å	3.5	0	100

## Data Availability

Data are available upon request.

## Supplementary Materials

The supporting information can be downloaded at https://www.mdpi.com/article/10.3390/ijms24021108/s1. References [25,34,40] are cited in the supplementary materials.
