# Supplementary material for: Structural Characterization of Murine Phosphodiesterase 5 Isoforms and Involvement of Cysteine Residues in Supramolecular Assembly
Source: Int J Mol Sci. 2023 Jan 6;24(2):1108. doi: 10.3390/ijms24021108 (PMC9862819; doi:10.3390/ijms24021108)

Supplementary material
Structural characterization of murine phosphodiesterase 5 isoforms and involvement of cysteine residues in supramolecular assembly.

Mauro Giorgi [1,*], Adriana Erica Miele [2,3,*], Silvia Cardarelli [1], Alessandra Giorgi [2], Mara Massimi [4], Stefano Biagioni [1] and Michele Saliola [1]

1 Department of Biology and Biotechnology “C. Darwin”, Sapienza University of Rome, Piazzale A. Moro 5 00185 Rome, Italy; silvia.cardarelli@uniroma1.it; stefano.biagioni@uniroma1.it; mauro.giorgi@uniroma1.it; michele.saliola@fondazione.uniroma1.it
2 Department of Biochemical Sciences, Sapienza University of Rome, Piazzale A. Moro 5 00185 Rome, Italy. alessandra.giorgi@uniroma1.it
3 UMR 5280 ISA - CNRS - UCBL, Université de Lyon, 5 Rue de La Doua, 69100 Villeurbanne, France; adriana.miele@univ-lyon1.fr
4 Department of Life, Health and Environmental Sciences, University of L’Aquila, Via Vetoio 67100 L’Aquila, Italy; mara.massimi@univaq.it

Supplementary Figure S1. Sequence coverage of MmPDE5A3 by MS after trypsin digestion. Residues in red are those covered by MS analysis. Cys residues were protected by iodoacetamide (IAM).

Supplementary Figure S2. Sequence coverage of MmPDE5A3 reacted with with N-ethyl-maleimide (NEM) by MS. Residues in red are those covered by MS analysis of tryptic peptides.

Supplementary Figure S3. Zoom of MS spectrum of the peptide 315-330 in MmPDE5A3 control and treated with NEM.

Supplementary Table S1. Localization of Cys residues mapped by MS on the homologous structures deposited in the PDB.

Supplementary Figure S1.

Sequence coverage of MmPDE5A3 by MS after trypsin digestion. Residues in red are those covered by MS analysis. The 25 Cys residues (yellow, bold, underlined) were protected by iodoacetamide (IAM). All the Cys, excepted 625 and 637, were visible. Cys 447, 522, 544 were oxidised to sulfenic acid.

MmPDE5A3 control

```
MVNAWFSERV HNIPVCKEGI RAHTESCSCS LQQSPHADNT TPGAPARKIS ASEFDRPLRP 60
IVVKDSEGTV SFLSDSGKKE QMPLTPPRFD SDEGDQCSRL LELVKDISSH LDVTALCHKI 120
FLHIHGLISA DRYSLFLVCE DSSKDKFLIS RLFDVAEGST LEEASNNCIR LEWNKGIVGH 180
VAAFGEPLNI KDAYEDPRFN AEVDQITGYK TQSILCMPIK NHREEVVGVA QAINKKSGNG 240
GTFTEKDEKD FAAYLAFCGI VLHNAQLYET SLLENKRNQV LLDLASLIFE EQQSLEVILK 300
KIAATIISFM QVQKCTIFIV DEDCPDSFSR VFHMECEEVG KPSDPLTREQ DANKINYMYA 360
QYVKNTMEPL NIPDVTKDKR FPWTNENMGH VNTPCIGSLL CTPIKNGKKN KVIGVCQLVN 420
KMEENTGKIK AFNQNDEQFL EAFVIFCGLG IQNTQMYEAV ERAMAKQMVT LEVLSYHASA 480
AEEETRELQA LSAAVVPSAQ TLKITDFSFS DFELSDLETA LCTIRMFTDL NLVQNFQMKH 540
EVLCRWILSV KKNYRKNVAY HNWRHAFNTA QCMFAALKAG KIQNKLTDLE TLALLIAALS 600
HDLDHRGVNN SYIQRSEHPL AQLYCHSIME HHHFDQCLMI LNSPGNQILS GLSIDEYKTT 660
LKIIKQAILA TDLALYIKRR GEFFELIRKN QFSFEDPLQK ELFLAMLMTA CDLSAITKPW 720
PIQQRIAELV AAEFFDQGDR ERKELNMEPA DLMNREKKNK IPSMQVGFID AICLQLYEAL 780
THVSEDCLPL LNGCRKNRQK WQALAEQQEK MLLNGESSQG KRD                   823
```

Supplementary Figure S2.

Sequence coverage of MmPDE5A3 reacted with N-ethyl-maleimide (NEM) by MS. Residues in red are those covered by MS analysis of tryptic peptides.

MmPDE5A3 + NEM

```
MVNAWFSERV HNIPVCKEGI RAHTESCSCS LQQSPHADNT TPGAPARKIS ASEFDRPLRP  60
IVVKDSEGTV SFLSDSGKKE QMPLTPPRFD SDEGDQCSRL LELVKDISSH LDVTALCHKI 120
FLHIHGLISA DRYSLFLVCE DSSKDKFLIS RLFDVAEGST LEEASNNCIR LEWNKGIVGH 180
VAAFGEPLNI KDAYEDPRFN AEVDQITGYK TQSILCMPIK NHREEVVGVA QAINKKSGNG 240
GTFTEKDEKD FAAYLAFCGI VLHNAQLYET SLLENKRNQV LLDLASLIFE EQQSLEVILK 300
KIAATIISFM QVQKCTIFIV DEDCPDSFSR VFHMECEEVG KPSDPLTREQ DANKINYMYA 360
QYVKNTMEPL NIPDVTKDKR FPWTNENMGH VNTPCIGSLL CTPIKNGKKN KVIGVCQLVN 420
KMEENTGKIK AFNQNDEQFL EAFVIFCGLG IQNTQMYEAV ERAMAKQMVT LEVLSYHASA 480
AEEETRELQA LSAAVVPSAQ TLKITDFSFS DFELSDLETA LCTIRMFTDL NLVQNFQMKH 540
EVLCRWILSV KKNYRKNVAY HNWRHAFNTA QCMFAALKAG KIQNKLTDLE TLALLIAALS 600
HDLDHRGVNN SYIQRSEHPL AQLYCHSIME HHHFDQCLMI LNSPGNQILS GLSIDEYKTT 660
LKIIKQAILA TDLALYIKRR GEFFELIRKN QFSFEDPLQK ELFLAMLMTA CDLSAITKPW 720
PIQQRIAELV AAEFFDQGDR ERKELNMEPA DLMNREKKNK IPSMQVGFID AICLQLYEAL 780
THVSEDCLPL LDGCRKNRQK WQALAEQQEK MLLNGESSQG KRD                    823
```

The long peptide (43 aa) containing Cys 625 and 637 was not visible neither in the control nor in the NEM-modified MmPDE5A3. Peptides with Cys highlighted in grey were not visible in the sample reacted with NEM.

Cys highlighted in cyan were not modified by NEM.

Cys highlighted in green are totally modified by NEM; Cys highlighted in yellow are partially modified by NEM; Cys highlighted in **black** were oxidized to sulfenic acid prior to react with NEM.

Table S1. Localization of Cys residues mapped by MS on the homologous structures deposited in the PDB. Localization of Cys within the regulatory (GAF-A and GAF-B) and catalytic (CAT) domains, based on the primary structure alignment of MmPDE5A3 (1-823) with the PDB deposited structures of MmPDE5A1 GAF-A (aa 154-302; PDB ID: 2k31 [1]), HsGAF-B (aa 346-508; PDB ID: 2xss [2]), HsCAT (aa 534-858; PDB ID: 5jo3) and HsPDE2, a cAMP phosphodiesterase (194-728, PDB ID: 3ibj [3]). NEM unequivocally modifies 3 Cys, one per domain, namely 258, 315, 711; it also modifies other 4 Cys, namely 117 on GAF-A, 447 on GAF-B, 522 and 544 in CAT. It is worth noticing that the last three Cys were already oxidised in the freshly purified sample prior to reaction with NEM, indicating a high reactivity despite their putative buried position (according to the homology structures).

Color code in Figure S2	Cys numbering according to MmPDE5A3	Position the homology structures
Cyan	16, 27, 29, 97	Possibly in GAF-A, not covered by any structure
**Black**	117	Possibly in GAF-A, Solvent exposed
Green	258	GAF-A, Solvent exposed
Yellow	139	GAF-A, Solvent exposed
Yellow	168	GAF-A, Solvent exposed
Grey	216	GAF-A, buried in a hydrophobic cavity
Green	315	GAF-B, buried
Cyan	324	GAF-B, solvent exposed
Yellow	336	GAF-B, solvent exposed
Grey	395	GAF-B, solvent exposed
Grey	401	GAF-B, buried
Grey	416	GAF-B, buried
**Black**	447 (sulfenic acid)	GAF-B, buried (same topological position as C216)
**Black**	522 (sulfenic acid)	CAT, buried (Ala in HsPDE2)
**Black**	544 (sulfenic acid)	CAT, buried (Ala in HsPDE2)
Grey	572	CAT, water filled cavity (Phe in HsPDE2)
invisible	625	CAT, solvent exposed (Asn in HsPDE5 and Arg in HsPDE2)
invisible	637	CAT, solvent exposed (Ala in HsPDE2)
Green	711	CAT, buried behind the catalytic Zn^{2+}
Grey	773	CAT, buried (Ala in HsPDE2)
Grey	787	CAT, buried (Ala in HsPDE2)
Grey	794	CAT, buried (Val in HsPDE2)

References:

1. Heikaus CC, Stout JR, Sekharan MR, et al. Solution structure of the cGMP binding GAF domain from phosphodiesterase 5: insights into nucleotide specificity, dimerization, and cGMP-dependent conformational change. *J Biol Chem*. 2008; **283**(33): 22749-22759. DOI: 10.1074/jbc.m801577200.
2. Russwurm M, Schlicker C, Weyand M, Koesling D, Steegborn C. Crystal structure of the GAF-B domain from human phosphodiesterase 5. *Proteins*. 2011; **79**(5): 1682-1687. DOI: 10.1002/prot.22989.
3. Pandit J, Forman MD, Fennell KF, Dillman KS, Menniti FS. Mechanism for the allosteric regulation of phosphodiesterase 2A deduced from the X-ray structure of a near full-length construct. *Proc Natl Acad Sci USA*. 2009; **106**(43): 18225-18230. DOI: 10.1073/pnas.0907635106.

Supplementary Figure S3. Zoom of MS spectrum which compares the peptide 315-330 between the control (top panel), where all the Cys are totally alkylated by IAM, and the NEM treated MmPDE5A3 (bottom panel), where Cys315 is alkylated by NEM and Cys324 is alkylated by IAM. The confirmation is derived from MSMS analysis.

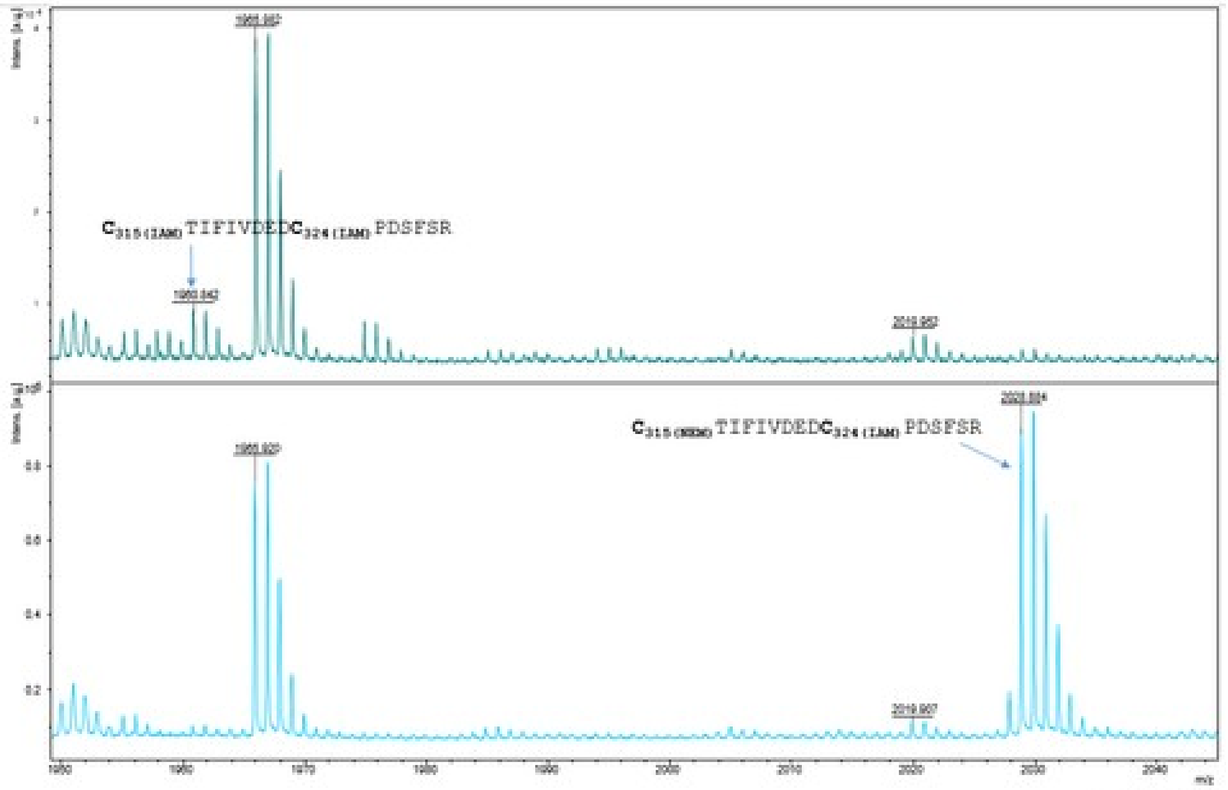

Supplement: Supplementary file 1 [file ijms-24-01108-s001.zip › ijms-2116741-supplementary.pdf]
